# Physiological Stress Mediated by Corticosterone Administration Alters Intestinal Bacterial Communities and Increases the Relative Abundance of *Clostridium perfringens* in the Small Intestine of Chickens

**DOI:** 10.3390/microorganisms8101518

**Published:** 2020-10-01

**Authors:** Sarah J. M. Zaytsoff, Richard R. E. Uwiera, G. Douglas Inglis

**Affiliations:** 1 Agriculture and Agri-Food Canada, Lethbridge, AB T1J 4B1, Canada; zaytsoff@ualberta.ca; 2 410 Agriculture/Forestry Centre, Department of Agricultural, Food, and Nutritional Science, University of Alberta, Edmonton, AB T6G 2P5, Canada; ruwiera@ualberta.ca

**Keywords:** physiological stress, microbiota, corticosterone, small intestine, cecum, *Clostridium perfringens*

## Abstract

A model of physiological stress mediated by the administration of corticosterone (CORT) was used to investigate the impact of stress on the intestinal microbiota of chickens. Birds were administered CORT in their drinking water at 0, 10 (low dose CORT; LDC), and 30 (high dose CORT; HDC) mg/L. Digesta from the small intestine and ceca were examined after 1, 5, and 12 days post-initiation of CORT administration by 16S rRNA gene sequencing. A decrease in phylogenetic diversity and altered composition of bacteria were observed for HDC in the small intestine. Analysis by ANOVA-Like Differential Expression 2 (ALDEx2) showed that densities of *Clostridium sensu stricto* 1 bacteria were increased in the small intestine for LDC and HDC. Quantitative PCR confirmed that CORT administration increased densities of *Clostridium perfringens* in the small intestine, but only HDC was associated with increased densities of the bacterium in ceca. Predictive functional analysis by Phylogenetic Investigation of Communities by Reconstruction of Unobserved States 2 (PICRUSt2) showed pathways of carbohydrate metabolism to be enriched with CORT, and amino acid synthesis to be enriched in control birds in the small intestine. In conclusion, physiological stress mediated by CORT modulated bacterial communities in the small intestine and increased densities of *C. perfringens*. This implicates stress as an important mediator of this important enteric pathogen in poultry.

## 1. Introduction

The activation of the hypothalamus–pituitary–adrenal axis in poultry results in the production of corticosterone (CORT), the primary glucocorticoid stress hormone. The production of CORT relays numerous impacts to bird health, including modifications to bird metabolism and immune function [1]. Impairment to weight gain and muscle assimilation are some of the reported effects of physiological stress on bird performance [2,3]. We have previously reported that birds treated with CORT promote hepatic lipid synthesis and distinctly alter metabolite profiles of liver, kidney, and breast muscle tissue [4]. It is well recognized that glucocorticoids impart many immunomodulatory effects on mammalian and avian species [5]. In poultry, it has been shown that short term CORT exposure can incite inflammatory responses, whereas chronic CORT exposure stimulates immunoregulatory responses [5]. The study of the intestinal microbiota of poultry and its relationship to host health and production performance is of great interest. Considering the modifications that physiological stress imparts on the host, and the bi-directional influence of the host–microorganism relationship, investigation of the poultry microbiota under physiological stress is warranted.

The intestinal microbiota has been shown to influence nutrition uptake, immune development, and provide colonization resistance against incoming intestinal pathogens [6,7]. Various factors can alter the composition of the microbiota in chickens, including the presence of antibiotics in feed, the age and sex of the animal, housing practices, and diet [8]. Modern poultry production can subject birds to varying stressful experiences and can result in negative health outcomes [1]. The influence of physiological stress on the poultry microbiota is beginning to be explored. For example, heat stress has been shown to affect the bacterial community composition of ileal and cecal digesta and feces in chickens [9,10,11]. Bacterial communities of the intestinal tract are sensitive to stress, and various alterations to the enteric environment can result in changes in the microbiota [12]. For example, social stress in mice altered the stability of the intestinal microbiota, reduced diversity, and promoted translocation of bacteria [13]. Physiological stress can alter the morphology of the intestine, promote digesta transit and mucus secretion, and alter mucosal permeability [12]. All of these parameters can influence the enteric environment and consequently disrupt the normal microbiota [12,14]. However, the mechanisms by which stress influences the microbiota and enteric disease remain speculative, particularly in chickens.

Alterations to the microbiota can affect host health and increase predisposition to enteric disease. Stress-induced modifications to the intestinal microbiota have been associated with increased cytokine production and the modulation of immune activity [13]. Putative beneficial bacteria (e.g., *Lactobacillus* spp.) have been shown to decrease in abundance following exposure to physiological stress [12,15]. Stress can also lead to increased colonization by foodborne pathogens such as *Escherichia coli* and *Salmonella* spp. [12,16]. Necrotic enteritis (NE) is a disease of the small intestine in chickens incited by *Clostridium perfringens*. Despite the manifestation of NE in the small intestine, afflicted birds can exhibit a perturbation of bacterial communities in ceca, although the mechanisms are unknown [17]. Notably, correlating enteric bacterial colonization patterns with disease may lead to the discovery of microbial biomarkers that may aid in the development of diagnostic methods and provide information on key factors that contribute to disease initiation and development.

Advances in DNA sequencing technology have promulgated an interest in defining how the microbiota may be modified within the intestine and influence host health. Given the ubiquitous nature of stress in poultry production, the current study examined how the enteric microbiota was altered in a chicken model of physiological stress mediated by the administration of the glucocorticoid stress hormone, CORT. We utilized this model of exogenous CORT administration as it consistently elevates levels of CORT and mediates a stress response in a prescribed manner [18]. Furthermore, this model has been used on numerous occasions to examine how stress alters host functions in poultry [18,19,20,21]. We hypothesized that the structure of bacterial communities in the small intestine and ceca of chickens administered CORT will be altered relative to the diversity and composition of the bacteria in birds not treated with CORT, and that resident populations of *C. perfringens* will be favored in birds with reduced bacterial diversity due to CORT administration. The objectives were to (1) incite physiological stress in chickens via administration of CORT; (2) measure the richness, composition, diversity, and structure of bacterial communities in the small intestine and ceca of birds ± CORT; and (3) ascertain the effects of physiologic stress on densities of the pathogen, *C. perfringens*.

## 2. Materials and Methods

### 2.1. Ethics Statement

This study was implemented in strict accordance with the recommendations specified in the Canadian Council on Animal Care Guidelines. The project study was reviewed and approved by the Lethbridge Research and Development Centre Animal Care Committee (Animal Use Protocol #1526) before the commencement of the research.

### 2.2. Study Design

This study was arranged as a completely randomized design with four levels of stress treatment and three levels of time (i.e., 4 × 3 factorial design with three biological replicates). The four stress treatments were: control (CON; untreated drinking water); ethanol carrier control (ECC; 0.2% ethanol in drinking water); low dose CORT (LDC; 10 mg CORT/L of drinking water); and high dose CORT (HDC; 30 mg CORT/L of drinking water). Birds were terminally sampled from each stress treatment at 1, 5, and 12 days after the initiation of CORT treatment (12 treatment groups, *n* = 3 per treatment). The three biological replicates were completed on separate occasions to ensure independence, and the experiment was comprised of 36 birds in total.

### 2.3. Animal Husbandry

Specific-pathogen-free white leghorn chickens eggs were obtained from the Canadian Food Inspection Agency (Ottawa, ON, CA). Eggs were incubated and hatched as previously described [4]. Chicks (1-day-old) were acclimatized in a group within one large animal pen for 10 days with free access to a brooder (Brinsea Products Inc., Titusville, FL, USA). At 11-days-of-age, birds were randomly assigned to the four stress treatments and housed in groups of four within an individually ventilated cage system (Techniplast, Montreal, QC, CA) as previously described [4]. Each animal cage contained an additional companion bird to ensure that no birds were left socially isolated. Birds were provided free access to a non-medicated starter diet (Hi-Pro Feeds, Lethbridge, AB, CA [22]) and water at all times. Birds were maintained on a 12 h light: 12 h dark cycle, and were weighed daily. CORT administration commenced in birds that were 14-days-of-age as described previously [4]. CORT (10 or 30 mg) was dissolved in 2.0 mL of absolute ethanol and added to 1 L of drinking water. Water containing CORT was prepared each morning and changed twice during each day.

### 2.4. Sample Collection 

One bird from each treatment was randomly selected at each sample time. Birds were anaesthetized with isoflurane (5% isoflurane; 1 L O_2_/min) and humanely euthanized by cervical dislocation. The small intestine and ceca were aseptically removed and longitudinally opened using a sterile blade. Digesta in the intestinal lumen was removed from the small intestine at the jejunum–ileum junction (Meckel’s diverticulum) and from ceca using a sterile wooden splint, and stored at −80 °C until processing.

### 2.5. DNA Extraction and 16S rRNA Gene Sequencing

Bacterial genomic DNA from intestinal digesta from the small intestine (jejunum–ileum) and ceca was extracted using a QIAamp Fast DNA Stool Mini Kit (Qiagen, Inc., Toronto, ON, Canada). DNA was sent to Genome Quebec (Montreal, QC, Canada) for library preparation and 16S rRNA gene sequencing. The V3-V4 region of the 16S rRNA gene was amplified using PCR primers 341F: 5′- CCTACGGGNGGWGCAG and 805R: 5′-GACTACHVGGGTATCTAATCC. The PCR product was then sequenced using a MiSeq (Illumina^®^, San Diego, CA, USA), obtaining 250 bp paired-end reads.

### 2.6. Sequencing Data Analysis

Quantitative Insights Into Microbial Ecology 2 (QIIME 2™, version 2019.10; [23]) was used to facilitate sequencing analysis while filtering of low quality reads (quality score <20), trimming sequences, and paired-end reads were joined using DADA2. Joined reads were grouped into exact amplicon sequence variants (ASVs, *n* = 1265) where taxonomy was classified using the SILVA bacteria reference database (release version 132) [24]. Low count reads, mitochondrial sequences, and chloroplast sequences were filtered out, and analysis was conducted on 1150 bacterial ASVs. Sampling depth for the small intestine and ceca was set to 15,000 and 23,000 reads, respectively. Three samples from the small intestine were omitted from analysis due to low sequence counts (1 from CON-Day 5 and 2 from LDC-Day 1 and 12). There was no difference among the sample times, and analysis of stress treatments was thus averaged over sample days. Core metric analysis was implemented in QIIME2 to obtain alpha (Faith’s phylogenetic and Shannon’s diversity) and beta (Jaccard, Bray–Curtis, and weighted UniFrac distance) diversity results. Alpha diversity was analyzed by pairwise comparisons of the Kruskal–Wallis test. Beta diversity was analyzed by pairwise permutational multivariate analysis of variance (PERMANOVA) [25]. A Benjamini and Hochberg correction was applied to both pairwise alpha and beta diversity tests when corrected *p*-values were <0.050 relative to both the CON and ECC treatments. A table of ASVs was exported from QIIME2 and used to generate compositional bar plots and heatmaps in GraphPad Prism (version 8.4.2). The R package ALDEx2 plugin was utilized in QIIME2 to differentiate significant taxa (*q* < 0.10) between control birds (CON and ECC groups) and CORT treatment birds (LDC and HDC) [26]. Percent abundance of significant taxa (*Clostridium sensu stricto* 1) was normalized, and one-way ANOVA was applied using the GraphPad Prism software (La Jolla, CA, USA, version 8.2.4) with a multiple comparison Tukey’s significant difference test. Results (LDC and HDC) were deemed significant when *p* < 0.050 relative to CON and ECC. Data in figures were plotted as mean ± standard error of mean (SEM). Predictive functional analysis was completed by using the PICRUSt2 plugin for QIIME2 [27]. The generated Kyoto Encyclopedia of Genes and Genomes (KEGG) ortholog and MetaCyc pathway abundance tables were analyzed by ALDEx2 to identify significant features that differed between the control treatment birds (CON and ECC) and those administered CORT (LDC and HDC).

### 2.7. Quantitative PCR

The basic quantitative PCR (qPCR) protocol described by Zaytsoff et al. [22] was used. Bacterial genomic DNA from the small intestinal and cecal digesta was extracted as indicated above. For standard curve generation, genomic DNA from a pure culture of *C. perfringens* was extracted using DNeasy Blood and Tissue Kit (Qiagen, Inc., Toronto, ON, Canada). Briefly, cell biomass was lysed using enzymatic lysis buffer (20 mM Tris⋅Cl, pH 8.0; 2 mM sodium EDTA; 1.2% Triton X-100; 20 mg/mL lysozyme) instead of the tissue lysis solution (Qiagen, Inc., Toronto, ON, Canada, Buffer ATL) supplied in the kit. A standard curve of known copies of 16S rDNA specific to *C. perfringens* was generated with DNA amplified from the extracted DNA using CP1.2 primers F: 5′-AAAGATGGCATCATCATTCAAC and R: 5′-TACCGTCATTATCTTCCCCAAA [28]. Amplicons were visualized in a 2% agarose gel, and the amplicon was extracted using a QIAquick Gel Extraction Kit (Qiagen, Inc., Toronto, ON, Canada). To generate a standard curve of known gene copies, the gel-extracted DNA was quantified fluorometrically using Qubit™ 2 (Life Technologies, Burlington, ON, Canada), and copies of genes were normalized to 10^7^ copies/µL based on concentration, amplicon size, and nucleotide weight. A standard curve was generated by diluting DNA in a 10-fold dilution series and amplifying *C. perfringens* 16S rDNA using CP1.2 primers. Quantitative PCR was used to measure *C. perfringens* densities in the small intestinal and cecal digesta relative to the standard curve and normalized by the weight of the sample. Each reaction contained 5.0 µL Quantitect SYBR green master mix (Qiagen, Inc., Toronto, ON, Canada), 0.5 µL of each primer (10 µM), 1.0 µL bovine serum albumin (1 mg/mL), 2.0 µL DNase-free water, and 1.0 µL template DNA. Reactions conditions were: 95 °C for 15 min; and 40 cycles of 95 °C for 15 s, 55 °C for 30 s, and 72 °C for 30 s; and melt curve analysis from 55–95 °C. An ABI7900HT thermocycler (Applied Biosystems, Carlsbad, CA, USA) was used. Reactions were run in triplicate, and the mean of the three observations was calculated. The qPCR results were assessed for normality using GraphPad Prism software (La Jolla, CA, USA, version 8.4.2), and analyzed by one-way ANOVA to determine differences among CORT treatments. A multiple comparison Tukey’s significant difference test was applied; results (LDC and HDC) with *p* < 0.050 were considered significant relative to both CON and ECC. Data are represented as mean ± SEM. 

### 2.8. Characterization of C. perfringens

Fecal samples were collected from birds at 16-days-of-age, serially diluted, and the suspension spread on Columbia agar supplemented with 10% sheep’s blood (Difco, Frankon Lakes, NJ, USA). Putative colonies for *C. perfringens* were re-streaked for biomass and extracted using a DNeasy Blood and Tissue Kit (Qiagen, Inc., Toronto, ON, Canada) as described above. Putative *C. perfringens* isolates were subjected to PCR using CP1.2 primers and netB toxin gene primers (F: 5′-AAATATACTTCTAGTGATACCGCTTCACA-3′; R: 5′-GAGGATCTTCAATAAATGTTCCACTTAA-3′) [29]. Reactions conditions were: 95 °C for 10 min; and 35 cycles of 95 °C for 15 s, 55 °C for 30 s, and 72 °C for 30 s. PCR products were visualized on a 2% agarose gel to identify strains that were ± *C. perfringens* and/or NetB positive.

### 2.9. Data Availability

The raw sequencing reads were submitted to the Sequencing Read Archive of NCBI under BioProject accession PRJNA647907. Sample metadata can be found in Supplementary Materials File 1.

## 3. Results

### 3.1. Corticosterone Treatment Alters the Intestinal Microbiota Composition

**Figure 1:**
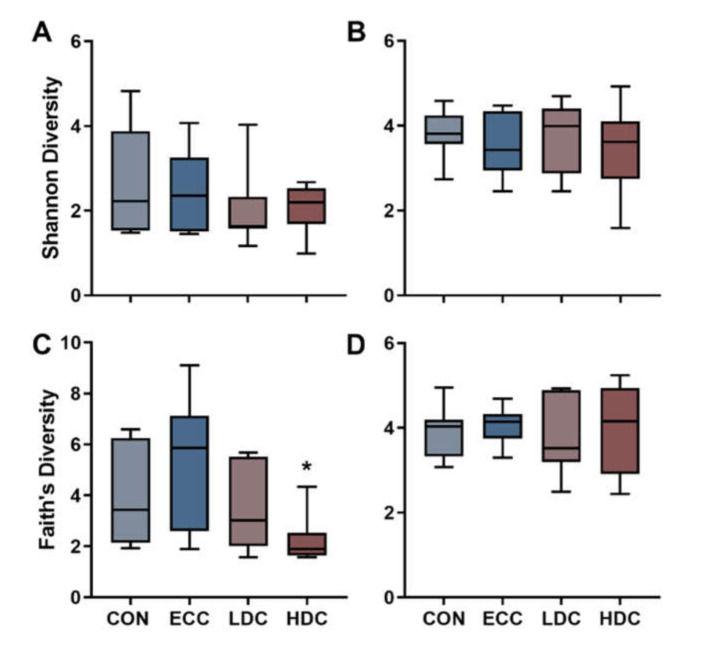
Effect of corticosterone administration on α-diversity of bacteria in the small intestine and ceca of chickens. Birds were administered normal drinking water (CON), 0.2% ethanol drinking water (ECC), 10 mg/L CORT (LDC), or 30 mg/L CORT (HDC). Shannon’s diversity in the (**A**) small intestine and (**B**) ceca. Faith’s phylogenetic diversity of the (**C**) small intestine and (**D**) ceca. * Indicates *p* < 0.050 in comparison to CON and ECC treatments.

**Figure 2:**
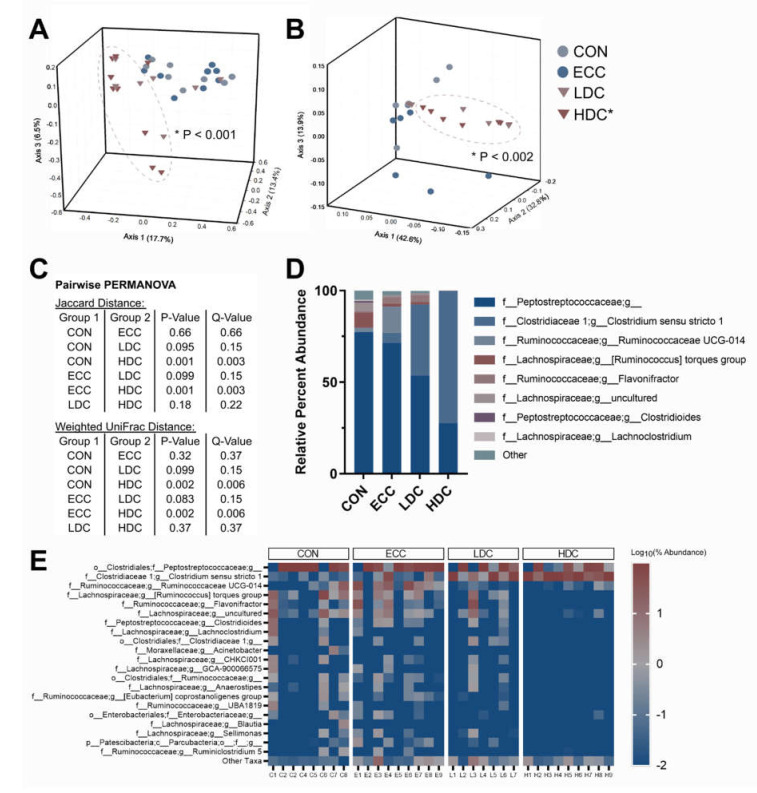
Corticosterone administration alters bacterial composition in the small intestine of chickens. Birds were administered normal drinking water (CON), 0.2% ethanol drinking water (ECC), 10 mg/L CORT (LDC), or 30 mg/L CORT (HDC). (**A**) Jaccard distance and (**B**) weighted UniFrac PCoA plots of the small intestine. Ellipsoids cluster around HDC treatment. (**C**) Pairwise PERMANOVA results for Jaccard and weighted UniFrac distance. (**D**) Percent abundance of the eight most abundant taxa in the small intestine. (**E**) Heatmap of top twenty-one taxa in the small intestine. *n* = 8 for CON, *n* = 9 for ECC and HDC, *n* = 7 for the LDC treatment. * Indicates that the HDC differed (*p* ≤ 0.002) from the CON and ECC treatments.

**Figure 3:**
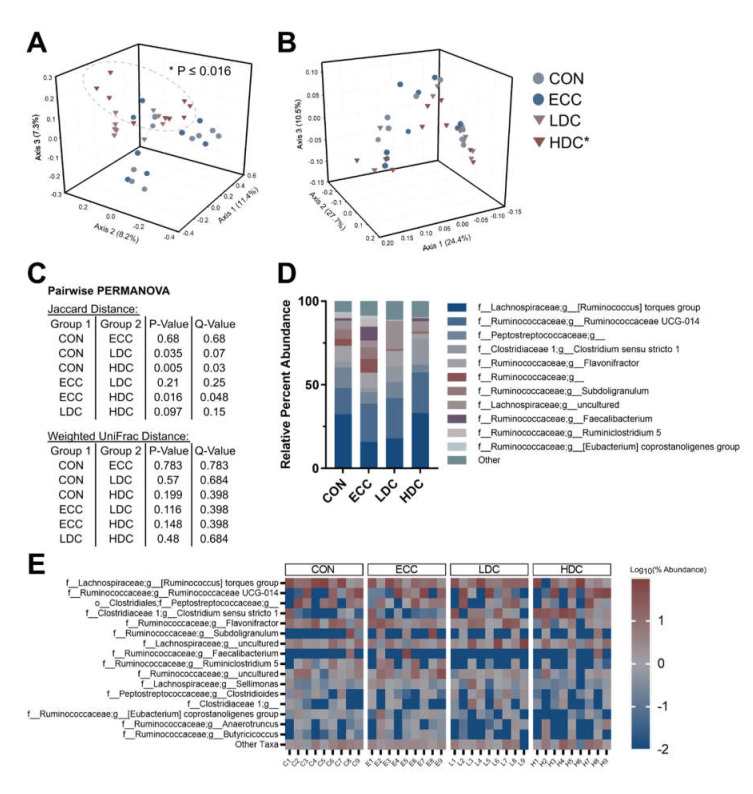
High dose corticosterone qualitatively modulates the bacterial communities in ceca of chickens. Birds were administered normal drinking water (CON), 0.2% ethanol drinking water (ECC), 10 mg/L CORT (LDC), or 30 mg/L CORT (HDC). (**A**) Jaccard distance and (**B**) weighted UniFrac PCoA plots of cecal bacterial communities. Ellipsoids cluster around HDC treatment. (**C**) Pairwise PERMANOVA results for Jaccard and weighted UniFrac distance. (**D**) Percent abundance of top eleven most abundant taxa in ceca. (**E**) Heatmap of top sixteen taxa in ceca. *n* = 9 for all treatments. * Indicates that the HDC differed (*p* ≤ 0.016) from the CON and ECC treatments.

Alpha diversity was assessed through Shannon’s and Faith’s phylogenetic diversity indices in the small intestine and ceca. No changes (*p* ≥ 0.240) in Shannon’s diversity were observed in the small intestine or ceca (Figure 1A,B). Faith’s phylogenetic diversity decreased in the small intestine (*p* ≤ 0.028) but not in ceca (*p* ≥ 0.760) of HDC treatment birds in comparison to the CON and ECC treatments (Figure 1C,D). No changes (*p* ≥ 0.150) in Faith’s phylogenetic diversity were observed in the small intestine and ceca with LDC treatment relative to CON and ECC treatment birds. Community similarity was evaluated by Jaccard, Bray–Curtis, and weighted UniFrac distances. The HDC treatment in the small intestine diverged from both CON and ECC treatments with Jaccard (*p* ≤ 0.003), Bray–Curtis (*p* ≤ 0.030), and weighted UniFrac (*p* ≤ 0.006) distances (Figure 2A–C). Sequences of the *Clostridium sensu stricto* 1 genus were higher in LDC and HDC treatment birds (Figure 2D). Heatmap visualization of ASVs in the small intestine showed extensive loss of taxa within the HDC treatment birds (Figure 2E). In ceca, bacterial community similarity was changed with HDC treatment relative to CON and ECC treatments, as measured by Jaccard’s (*p* ≤ 0.048) (Figure 3A). Bray–Curtis (*p* ≥ 0.102) and weighted UniFrac (*p* ≥ 0.120) distance did not demonstrate any compositional changes among treatments (Figure 3B,C). Bacteria within the cecal digesta possessed a relatively stable taxonomic distribution, and no single taxon dominated among any of the treatments (Figure 3D,E).

### 3.2. Corticosterone Treatment Increases C. perfringens Densities

**Figure 4:**
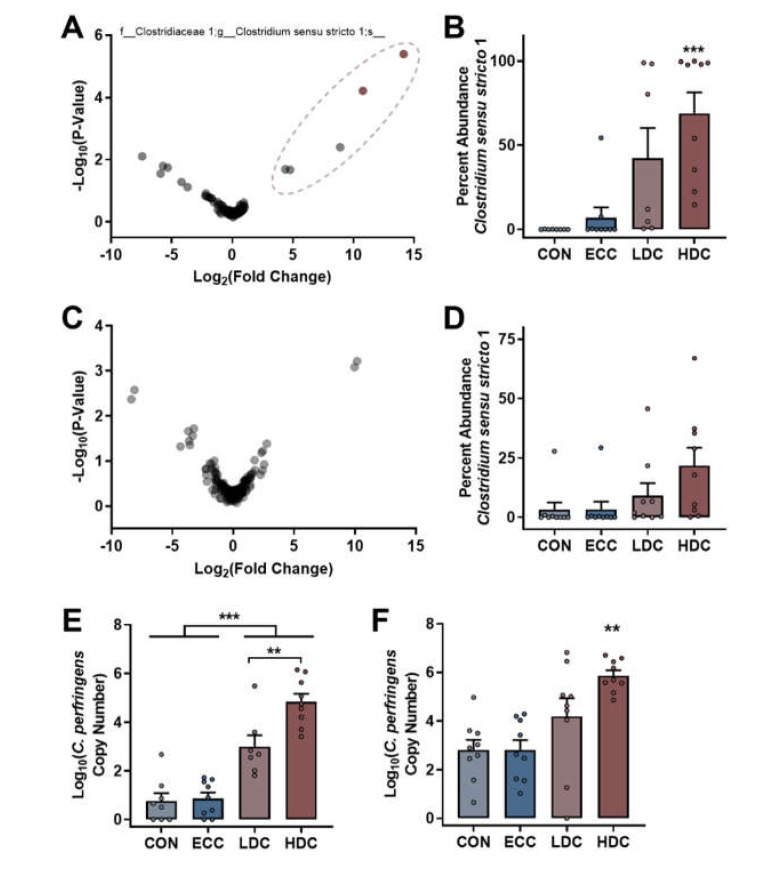
ALDEx2 analysis and qPCR determine that *C. perfringens* densities increase in the small intestine and ceca of chickens administered CORT treatment. Birds were administered normal drinking water (CON), 0.2% ethanol drinking water (ECC), 10 mg/L CORT (LDC), or 30 mg/L CORT (HDC). (**A** and **C**) Volcano plot constructed from the ALDEx2 output of (**A**) small intestine and (**C**) ceca. ASVs within the ellipsoid were identified as *Clostridium sensu stricto* 1, and significant ASVs are shown in pale red. (**B** and **D**) Percent abundance of *Clostridium sensu stricto* 1 sequences in the (**B**) small intestine and (**D**) ceca. (**E**–**F**) qPCR analysis of *Clostridium perfringens* in the (**E**) small intestine and (**F**) ceca. ** Indicates *p* < 0.010, and *** indicates *p* < 0.001 in comparison to the CON and ECC treatments.

ALDEx2 analysis was applied to both the small intestine and cecal bacterial community data to identify taxa that differed between non-CORT treatment (CON and ECC) and CORT-treatment (LDC and HDC) birds. Analysis of small intestinal communities identified two ASVs that were both classified in the genus *Clostridium sensu stricto* 1 (Figure 4A). The abundance of *Clostridium sensu stricto* 1 ASVs in LDC (*p* ≤ 0.015) and HDC (*p* < 0.001) treatment birds was higher than CON and ECC treatment birds (Figure 4B). In contrast to the small intestine, no ASVs were determined to be altered by ALDEx2 analysis in ceca (Figure 4C). However, the abundance of *Clostridium sensu stricto* 1 ASVs in ceca was higher (*p* ≤ 0.017) in HDC treatment birds in comparison to CON and ECC treatment birds (Figure 4D). The Basic Local Alignment Search Tool (BLAST) analysis of the two ASVs that were more abundant in the small intestine of LDC and HDC treatment birds by ALDEx2 analysis revealed a strong sequence match (99.76% and 100%) with *C. perfringens*. Furthermore, several isolates recovered from feces were identified as *C. perfringens* by PCR; no isolates were positive for the netB toxin gene. Thus, qPCR was performed on DNA extracted from small intestinal and cecal digesta. Densities of *C. perfringens* determined by qPCR corresponded to Illumina sequencing results. In this regard, the small intestine contained higher densities (*p* < 0.001) of *C. perfringens* in CORT treatment birds (both LDC and HDC) in comparison to birds not administered CORT (CON and ECC; Figure 4E). Moreover, only the HDC treatment birds showed higher densities (*p* < 0.001) of *C. perfringens* in ceca (Figure 4F).

### 3.3. Predictive Functional Analysis Is Altered with Corticosterone Administration

**Figure 5:**
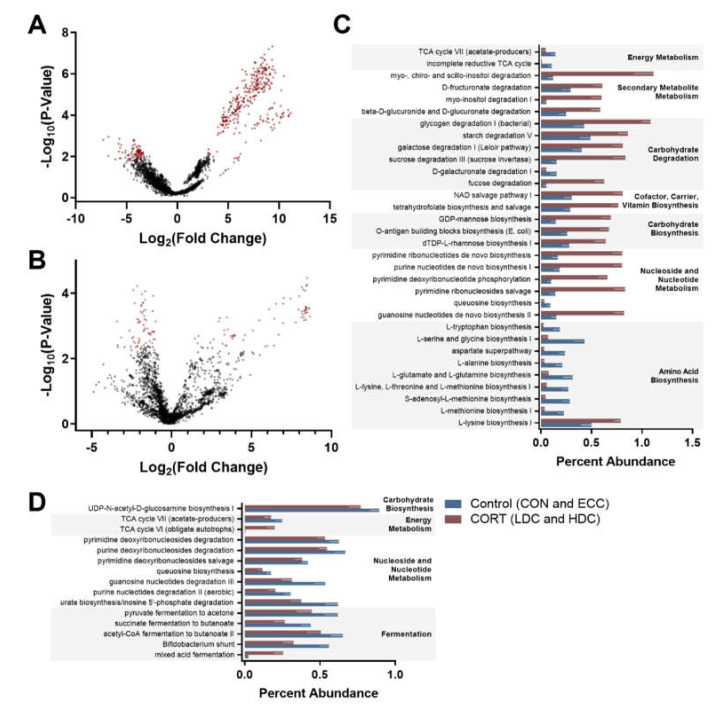
ANOVA-Like Differential Expression 2 (ALDEx2) analysis of Phylogenetic Investigation of Communities by Reconstruction of Unobserved States 2 (PICRUSt2) output with a MetaCyc pathway abundance in the small intestine and ceca of chickens. Birds were administered normal drinking water (CON), 0.2% ethanol drinking water (ECC), 10 mg/L CORT (LDC), or 30 mg/L CORT (HDC). (**A** and **B**) Volcano plot constructed from the ALDEx2 output of (**A**) small intestine and (**B**) ceca. Significant Kyoto Encyclopedia of Genes and Genomes (KEGG) ortholog metagenome predictions are shown in red. (**C** and **D**) Percent abundance of MetaCyc pathway predictions determined as significant by ALDEx2 analysis for (**C**) small intestine and (**D**) ceca.

Predictive functional outputs (KEGG Orthologs (KOs) and MetaCyc pathway abundance) from PICRUSt2 were analyzed using ALDEx2 to identify significant features among control (CON and ECC) and CORT treatment (LDC and HDC) birds. ALDEx2 analysis identified 430 and 125 KOs that were altered between control and CORT treatment birds in the small intestine and ceca, respectively (Figure 5A,B). Pathway abundance in the small intestine differed for 32 pathways (Figure 5C). In general, carbohydrate biosynthesis and degradation were enriched in CORT administered birds, while amino acid biosynthesis was more abundant in control birds. ALDEx2 analysis of pathway abundance in ceca identified 15 pathways that were altered between the control and CORT treatment birds (Figure 5D).

## 4. Discussion

The primary objective of the current study was to understand the degree to which bacterial communities are altered in the small intestine and ceca of chickens when subjected to physiological stress mediated by CORT administration. We observed that the microbiota in the small intestine was more sensitive to CORT-induced changes, and both the diversity and composition of bacterial communities were affected. The microbiota in ceca was affected by CORT administration to a lesser degree than in the small intestine, although qualitative changes in the composition of the bacterial community were observed. Significantly, bacterial sequences identified as *Clostridium sensu stricto* 1 increased in the small intestine of birds administered CORT, and qPCR analysis indicated that the increase in *Clostridium sensu stricto* 1 sequences was attributed to *C. perfringens*, the incitant of NE in chickens. 

The high bacterial richness in the intestine is associated with positive health benefits, and a functionally diverse microbiota is better able to occupy niches inhabited by pathogens [30]. There are many factors that can influence bacterial richness in chickens, especially when accounting for the variability in bacterial development in young birds [8,31]. We observed a decrease in intra-bird phylogenetic diversity with the HDC treatment in the small intestine. The induction of a social stressor in mice resulted in the alpha diversity of bacterial communities in ceca to be decreased [13]. Likewise, social conflict in wild male birds was associated with reduced bacterial phylogenetic diversity in fecal samples [32]. A reduction in diversity may be due to innate immune activation as a result of acute stimulation of the hypothalamic–pituitary–adrenal axis [33]. In chickens, short-term CORT administration has been shown to increase the expression of some inflammatory cytokine and chemokines [5,34]. The modulation of an immune response by CORT, taken together with the instability of the microbiota in young birds, may have contributed to a reduction in diversity that was observed in HDC birds. Moreover, the overgrowth of *C. perfringens* may have hindered the restoration of bacterial diversity.

In the present study, we observed that bacterial communities in the small intestine of chickens administered HDC clustered separately from other treatments. In ceca, we also observed qualitative changes in bacterial communities in birds administered HDC. Our results correspond with previous reports that demonstrated that bacterial communities were affected by high temperatures during the rearing of broiler and layer chickens [9,11]. The host–microorganism relationship is complex and functions in a bi-directional manner. It is uncertain whether stress altered the host in a manner that alters bacterial communities or the modulations to bacterial communities, consequently, modified the host. Various factors induced by stress may be driving changes in the microbiota. Intestinal morphology can be altered by heat stress, including decreased crypt depth [12]. The integrity of the intestinal structure is imperative to maintain barrier function and prevent the translocation of bacteria [12,13]. A study investigating the impacts of a social stressor in mice showed that bacteria could translocate to secondary lymphoid tissue under conditions of stress [35]. The intestinal mucus barrier is another factor that can be modulated by physiological stress. Intestinal glycans have been shown to regulate bacterial communities and limit the number of microorganisms that access the epithelium [36]. We have previously shown that stress induced by CORT increased mucin quantities in the small intestine of chickens [22]. Additionally, we have demonstrated that *C. perfringens* colonization in the presence and absence of CORT administration could modify the composition of intestinal mucus glycans [22,37]. This verifies that bacteria, and likely, the shift in bacterial communities, can result in changes in the host. We have also previously reported that CORT administration can alter the expression of tight junction proteins in the small intestine, as well as immune cytokines in the spleen and thymus [22]. Although our previous work did not examine the microbiota, it is likely that changes in the intestinal bacterial community occurred as was observed in the current study, and this may have modulated host functions.

The microbiota in the small intestine was affected to a greater extent than in ceca of birds administered CORT. This may be due to the substantially higher diversity of bacteria within ceca. In general, chickens have decreased bacterial diversity in the intestine in comparison to other species [38]. Although the reason for this is not fully understood, it has been attributed to the relatively fast transit time of digesta in chickens [38]. Our study confirmed a substantially higher alpha diversity in ceca in comparison to the small intestine. Bacterial diversity is an important driver of immune development, and delayed colonization by commensal bacteria or decreased bacterial diversity due to stress can permanently alter T cell receptor repertoires and, consequently, result in aberrant immune reactions to commensal microbiota [39]. These concepts enforce the significance of promoting a diverse microbiota to aid in host health. It is noteworthy that the current study was coordinated in a research facility with high hygiene standards, and birds thus had limited exposure to microorganisms from the environment. The maintenance of birds in the research facility likely contributed to the low abundance of *Lactobacillus* spp. that we observed, which are considered to be a key group of bacteria in the small intestine of poultry [8]. Moreover, study findings accentuate the importance of exposing birds to microorganisms after hatching to expedite enteric bacterial diversity, stability, and colonization resistance. 

CORT administration significantly affected the abundance of bacteria within the genus, *Clostridium sensu stricto* 1, to which *C. perfringens* belongs. Considering the importance of NE incited by *C. perfringens* on poultry production worldwide, we used qPCR to examine the impacts of stress on *C. perfringens* densities. In this regard, CORT administration was associated with increased densities of *C. perfringens* in the small intestine and ceca (only with HDC treatment). *Clostridium perfringens* is an endospore-forming taxon, so it is not unexpected that the birds became colonized with the bacterium via the ingestion of substrates during hatching and present within the rearing environment [40,41]. Birds were not administered any antimicrobials in the current study, which may have been a contributing factor to *C. perfringens* overgrowth. For example, chickens on organic farms not administered antimicrobials exhibit higher colonization rates by *C. perfringens* [8,42]. Our results are in agreement with other studies in poultry that have demonstrated that potential stressors experienced during production, such as heat and cold temperature exposures and high stocking densities, can be associated with increased densities of *C. perfringens* [43,44,45]. It is unclear from the results of the current study, as well as previous reports, whether increased *C. perfringens* densities may have been a contributor to altered bacterial diversity observed in the current study. Previous reports linking stress with increased *C. perfringens* densities did not examine the intestinal microbiota [43,44,45]. The altered structure of bacterial communities that we observed, which coincided with increased *C. perfringens,* are not mutually exclusive factors as *C. perfringens* only increased in birds receiving CORT treatment. In the absence of stress, *C. perfringens* infection has previously been shown to affect the cecal microbiota [17]. It is noteworthy that birds in the current study did not show any overt signs of infection consistent with NE. Additionally, the *C. perfringens* identified in our study was not positive for the netB toxin gene. As the NetB toxin is considered to be an important virulence factor, it is unlikely that the *C. perfringens* present in the intestines of chickens in the current study would incite NE [46]. However, as NetB positive and NetB negative bacteria exhibit similar isolation and colonization patterns in chickens [47], it is expected that NetB positive isolates would respond similarly to NetB negative isolates under conditions of physiological stress. This warrants investigation in future studies. 

Predictive functional analysis can provide potential insights into bacterial function. In general, metabolic pathways related to carbohydrate biosynthesis and degradation were predicted to be enriched in the small intestine of birds administered CORT. Conversely, amino acid biosynthesis pathways were predicted to be enriched in the small intestine of control birds. The disproportionately high abundance of *C. perfringens* in CORT-treated birds may account for why some predictive functions were altered between control and CORT treatment birds. For example, *C. perfringens* harbors several carbohydrate-active enzymes that can function to degrade glycans of intestinal mucus [37]. Furthermore, a virulent *C. perfringens* strain (i.e., CP1) has been shown to utilize sialic acid of chicken intestinal mucus and may be a contributing function in the development of NE in poultry [48]. A high protein diet has been proposed as a predisposing factor to *C. perfringens* overgrowth, as the bacterium lacks genes for several amino acid synthesis pathways [49,50]. The utilization of dietary protein by *C. perfringens* may reduce the host’s capacity to assimilate amino acids in an overgrowth state of *C. perfringens,* and potentially render fewer resources for weight gain. We have previously observed impaired weight gain in birds administered CORT at the doses used in the current study, and also observed reduced weight gain in the current study (Supplementary Materials File 2) [4]. The relationship among intestinal communities, bird metabolism, and health in the context of bacterial metabolism warrants study. Future research could include prescribed metabolite measures or metabolomics in birds challenged with *C. perfringens* ± stress to provide insights into bacterial functions and potential benefits or consequences to host health. 

In conclusion, we demonstrated that controlled physiological stress mediated by CORT administration altered bacterial communities in the small intestine, including an increase in the density of *C. perfringens.* Predictive functional analysis identified possible modulations to bacterial function following CORT administration. Given that stress can modulate a variety of host functions, including metabolism, future studies should implement a multi-omics approach to better understand the interactions between the host and the microbiota during their development in chickens, and importantly, examine how this relationship evolves over time under conditions of physiological stress. As birds were not exposed to any antimicrobials in the current study (i.e., as a confounding effect), study findings implicated physiological stress as an important mediator of the microbiota, including *C. perfringens*, and supported stress as a predisposing factor to NE. Future research should include challenging birds with both stress and a known virulent strain of *C. perfringens* to ascertain the mechanisms by which stress predisposes birds to NE. Lastly, deciphering interactions between hosts ± stress and the microbiota will be beneficial to developing novel, non-antibiotic, and tailored strategies in poultry production.

## Supplementary Materials

The following are available online at https://www.mdpi.com/2076-2607/8/10/1518/s1, Supplementary Materials File 1: Project Metadata. Supplementary Materials File 2: Bird Body Weight Gain.
