# Supplementary material for: Physiological Stress Mediated by Corticosterone Administration Alters Intestinal Bacterial Communities and Increases the Relative Abundance of Clostridium perfringens in the Small Intestine of Chickens
Source: Microorganisms. 2020 Oct 1;8(10):1518. doi: 10.3390/microorganisms8101518 (PMC7650536; doi:10.3390/microorganisms8101518)
Supplement: Supplementary file 1 [file microorganisms-08-01518-s001.zip › Supplementary Files_Microorganisms 920394/Supplemental File 2_Microorganisms 920394.docx]

Physiological Stress Mediated by Corticosterone Administration Alters Intestinal Bacterial Communities and Increases the Relative Abundance of *Clostridium perfringens* in the Small Intestine of Chickens

Sarah J. M. Zaytsoff ^1,2^, Richard R. E. Uwiera ^2^ and G. Douglas Inglis ^1,^*

**Supplemental Table 2.** Bird Body Weight Gain^#^.

|  | Average Cumulative Weight Gain (g) | | |
| --- | --- | --- | --- |
|  | Day 0 to Day 1 n=9 | Day 1 to Day 5 n=6 | Day 5 to Day 12 n=3 |
| CON | 10.7 | 45.2 | 87.1 |
| ECC | 9.6 | 44.4 | 76.5 |
| LDC | 4.7** | 16*** | 44.7* |
| HDC | 4.9** | 8.6*** | 48.4* |

* P < 0.05; ** P < 0.01: *** P < 0.0001 in comparison to CON and ECC treatments

# Data has been previously published in a different format. Bird body weight has been previously reported [1]. This data represents body weight gain.

1. Zaytsoff, S.J.M.; Brown, C.L.J.; Montina, T.; Metz, G.A.S.; Abbott, D.W.; Uwiera, R.R.E.; Inglis, G.D. Corticosterone-mediated physiological stress modulates hepatic lipid metabolism, metabolite profiles, and systemic responses in chickens. *Sci Rep* **2019**, *9*, 19225, doi:10.1038/s41598-019-52267-6.
